# AADOCR: Science First and Collective, Joyful Effervescence

**DOI:** 10.1177/00220345221096610

**Published:** 2022-06-08

**Authors:** J.A. Weintraub

**Affiliations:** 1 Adams School of Dentistry, University of North Carolina, Chapel Hill, NC, USA

**Keywords:** COVID-19, evidence-based dentistry, quality of life, self-renewal, stress, oral health

## AADOCR President-Elect Address, March 23, 2022

Esteemed leaders, distinguished guests, members, and friends.

Welcome and thank you, both to those who are here in Atlanta, Georgia, and those joining us online for our first hybrid annual AADOCR meeting. I am joyful as we celebrate coming together to share our science.

If you are in the audience, here or virtually, and you are a new member or first-time attendee, please stand if you are able, so we may welcome you. Please join the group standing if you are able, if you are participating in any role, directly or indirectly, in this meeting, if you have served in a participatory role in the past or plan to in the future. [Note: Almost everyone in the auditorium stood up.] Let’s thank each other for making this a great, participatory organization.

We know that the pandemic has taken a toll on the world. We extend our condolences to those who have lost loved ones. Congratulations to those who have been through severe illness and made it through.

It has been a challenging and stressful time for the research community. For a while, some couldn’t get to their labs. Clinical researchers couldn’t get to their study participants. Others had or still have funding delays and supply-side slowdowns, delaying arrival of needed supplies and equipment. Productivity was adversely affected for those working at home while caring for children and other family members. Yet, we continue to persevere to overcome challenges.

Our activities reflect our AADOCR’s new byline, “science first,” reflecting our shared language, passion, and principles. What does science first mean? As a *Star Trek* fan in the sixties, one aspect of science meant, paraphrased, “to seek out new life and new civilizations, to boldly go where no (human) has gone before.”

For me, part of the beauty of science is that it continually evolves as we seek the truth. As a result, the recommendations for how to apply the science also change. It is an iterative process. New knowledge can make us readjust prior thinking and change our behaviors and research methods. Advances in science require dialogue and debate. It is hard when new findings oppose long-held beliefs. Conflict may precede consensus. We make mistakes, face setbacks, reflect, and try again.

Unfortunately, we have seen science be under attack in our country with the spread of polarizing misinformation on social media. We know science is based on data and facts. As scientists, we do our best to be objective and minimize bias. As humans, we have flaws and are subject to bias. We try to acknowledge the limitations of our work as best we can. The next study can improve on the last one.

During this pandemic, we have seen great leaps in science with the advancement of new vaccines. We also know that they are based on the cumulative efforts of years of careful laboratory work. Within our own disciplines, there has been an explosion of COVID-related articles in our *Journal of Dental Research*, the *JDRCTR*, and in reports being presented this week.

It has been a time of innovation and opportunities. We have embraced new ways of sharing, communicating, and collaborating, from our new AADOCR website and community board feature, more webinars, and now this first, hybrid meeting.

I am passionate about what we do. We are striving to increase our understanding about how biology works, how it interacts with the internal and external environment, with social determinants of health, with human and organizational behavior, and how to apply it at the individual, system, or policy level to prevent disease and improve oral and overall health.

A *New York Times* article (Grant 2021) was titled, “There’s a specific kind of joy we’ve been missing.” It’s called “collective effervescence.” It’s what people feel when they come together for a shared purpose. We are participating in this meeting because of a shared purpose, our scientific mission, and our natural curiosity. As researchers, asking questions and learning new things can bring us joy. This week, it can be joyful, in person or via the online chat box, to meet new colleagues and renew friendships and share those eureka moments of scientific discovery. I look forward to meeting many of you. If you are not already an IADR member, I am inviting you to join and participate all year long. Our organization is here to help us support each other. The pandemic has been long and hard. Our CEO Christopher Fox and his fabulous team have worked hard to prepare for this meeting.

In closing, I extend my grateful thanks to my parents for valuing education, my husband for his love and support, and everyone who has mentored and collaborated with me along the way. Now is a time for sharing science and collective, joyful effervescence.
